# Prediction of potential suitable habitats in the 21st century and GAP analysis of priority conservation areas of *Chionanthus retusus* based on the MaxEnt and Marxan models

**DOI:** 10.3389/fpls.2024.1304121

**Published:** 2024-02-29

**Authors:** Yongji Wang, Kefan Wu, Ruxia Zhao, Liyuan Xie, Yifan Li, Guanghua Zhao, Fen-Guo Zhang

**Affiliations:** ^1^ School of Life Science, Shanxi Engineering Research Center of Microbial Application Technologies, Shanxi Normal University, Taiyuan, China; ^2^ Administrative Office, Shanwei Middle School, Shanwei, China

**Keywords:** *Chionanthus retusus*, climate change, Maxent model, ENMeval, Marxan model, habitat shift

## Abstract

*Chionanthus retusus (C. retusus)* has a high economic and medicinal value, but in recent years it has been included in the list of China's major protected plants and China's Red List of Biodiversity due to the serious destruction of its wild germplasm resources. Based on 131 sample points of *C. retusus*, this study simulated potential habitats and spatial changes of *C. retusus* in the 21st century using the Maxent model combined with the geographic information system ArcGIS, predicted prioritized protected areas by the Marxan model, and assessed current conservation status through GAP analysis. The results showed that (1) when the regularization multiplier was 1.5 and the feature combinations were linear, quadratic, and fragmented, the area under the curve of the subjects in the training and test sets were both above 0.9, the true skill statistic value was 0.80, and the maximum Kappa value was 0.62, meaning that the model had high accuracy; (2) Temperature seasonality, annual precipitation, min temperature for coldest month, and precipitation of wettest month had relatively strong influences on species' ranges. (3) The moderately and optimally suitable habitats of *C. retusus* were primly located in the areas of southwestern Shanxi, central Hebei, western Henan, Shandong, Shaanxi, Anhui and Hubei; (4) Under different future climate scenarios, the area of each class of suitable habitat will increase for varied amounts compared to the current period, with a general trend of expansion to the south; (5) The *C. retusus* priority protected areas were mainly located in most of Shandong, southern Liaoning, southwestern Shanxi, western Henan, and central Hebei, and its conservation vacancy area was relatively large compared to its protected area. These results will provide scientific strategies for implementing long-term conservation of *C. retusus* in China and similar regions under warming conditions in the 21st century.

## 1 Introduction

Climate is a significant environmental condition influencing the geographic distribution of species. Climate change all around the world affects the growth habits, morphological features, and potential suitable habitats of species (Doxford and Freckleton, 2012). Beginning in the 20th century, as a result of anthropogenic influence and natural disasters, the acceleration of global climate change impacts both temperature and precipitation, as well as ecological degradation, causing damage to the stability and equilibrium of the global ecosystem structure (Lambert et al., 2010). For this purpose, plants will change their phenology or growth habits in response and select fresh adaptive regions through a range shift. So far, exploring the impact of climate change on the potential habitats of species has been widely used in the protection of rare plant resources and the introduction and cultivation of economic plants and has become a hot direction for the study of the impact of global changes on species (Zhao Y. et al., 2021). Species distribution models (SDMs) based on the niche theory are crucial methods for predicting the potential suitable habitats of species (Evans et al., 2010). Among them, the maximum entropy model (MaxEnt), as a kind of SDM, uses data from the extant distribution sites of the species and its associated environmental variables to explore its suitable environmental conditions for survival and to simulate its potential suitable habitat within the research area.

Systematic conservation planning (SCP) is a commonly used method for identifying conservation priority areas (Margules and Pressey, 2007) by considering the size, connectivity, length of boundaries, and social and economic costs of establishing protected areas while achieving conservation of species and minimizing conservation costs (Zhang et al., 2015). With the development of research, there is more and more software for systematic conservation planning, such as Marxan, C-Plan, and Zonation (Bárbara et al., 2018; De Alban et al., 2021), and they are usually combined with a geographic approach to the protection of biological diversity to assess the protection gap of the priority areas of the species in the existing nature reserves, which provides important information for the construction of nature reserves and has been widely applied at global scales (Rodrigues et al., 2004a; Chi et al., 2017).

*Chionanthus retusus* Lindl. et Paxt. (*C. retusus*) is a deciduous shrub or small tree that is sun-loving and drought-resistant. Its growth rate is slow, but adaptable and long-lived, growing in China’s temperate and subtropical regions, distributed in sparse mixed forests or bushes. Its flowers, buds, leaves, and other parts are rich in medicinal ingredients (Wang et al., 2022a). As a renowned ornamental tree, it is known for its oval leaves and striking snow-white flowers, often planted along streets for decorative purposes. Its root system is developed, which can be used as a rootstock for grafting osmanthus, and its wood is strong and meticulous (Chien et al., 2004). It is due to the broad range of potential applications that *C. retusus* has been extensively excavated and transplanted in recent years. However, this has led to the disruption of the tree’s growth pattern and the severe depletion of its wild germplasm resources. Now, the majority of the research on *C. retusus* concentrates on its stress resistance, genetic diversity, environmental conditions required for seed germination, and reproduction and cultivation technology (Pei et al., 2020; Qu et al., 2020; Zhou et al., 2021; Wang et al., 2022a); however, research on the distribution of its current germplasm resources and the prediction of its suitable habitats under future climate change scenarios is lacking.

Therefore, we selected climatic data and other environmental data related to them such as soil and topographical variables for the prediction of suitable areas for *C. retusus* for current and future periods using the MaxEnt model optimized by the “ENMeval” package. The study aims to 1) predict the potential suitable habitat for *C. retusus* in China under current climatic conditions and explore the significant environmental variables that limit its distribution as well as the environmental conditions of suitable habitats; 2) predict potential suitable habitats based on scenarios of global climate change over the 21st century and explore how its spatial configuration changes under different climatic scenarios; and 3) use the Marxan model most widely applied in conservation planning, which is used to assess conservation priority areas and to assess the conservation effectiveness of *C. retusus* in combination with methods of vacancy analysis. The above findings will assist in the development and adaptation of policies for the conservation of *C. retusus* under future conditions, which is of significance for the recovery of its natural population and the introduction of artificial cultivation.

## 2 Materials and methods

### 2.1 Data compilation and processing

#### 2.1.1 Species distribution sample points

**Figure 1:**
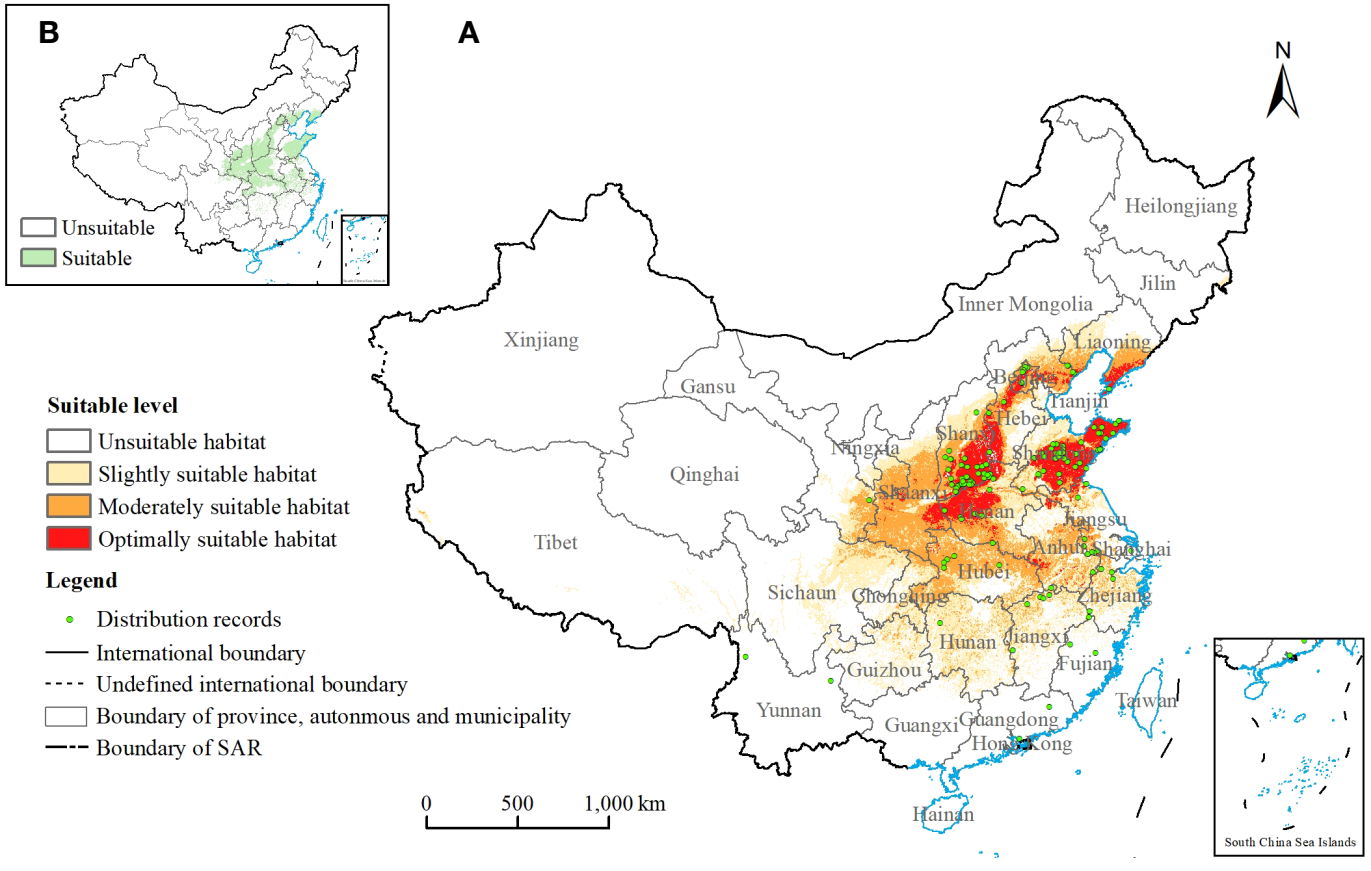
**(A)** Potential suitable habitats of *Chionanthus retusus* and distribution of record sites under current climatic conditions. **(B)** *Chionanthus retusus* suitable/unsuitable distribution maps for the current time period.

The Global Biodiversity Information Facility (GIBF, http://www.gbif.org), the National Herbarium Resource Centre (NSII, http://www.nsii.ac.cn), the Digital Herbarium of China (http://www.cvh.org.cn), and related literature (Qu, 2019; Tao, 2021) were searched for the occurrence records of *C. retusus*, and the information collected was used as distribution data. For the distribution data that were not labeled with precise geographic coordinates but indicated that the collection location was at the township or more precise level, their longitude and latitude were determined by querying their area name using Google Earth. We eliminated the wrong coordinate information and duplicate sample points, and only one point was kept for each 5 km × 5 km grid, ultimately obtaining the information of 131 effective distribution sample points of *C. retusus* in China (**Figure 1**), which was converted into.csv format for preservation.

#### 2.1.2 Environmental data

In this study, 38 environmental variables were initially selected (**Supplementary Table S1**). Among them, 19 bioclimatic variables were taken from WorldClim version 2.1 (http://www.worldclim.org), and the spatial resolution was 2.5 arc minutes; 16 soil variables were taken from the Food and Agriculture Organization of the United Nations World Soil Database (http://www.fao.org); and there were three topographic variables at a resolution of 30 m, where the elevation data were derived from the Digital Elevation Model dataset, and the slope and aspect data were both extracted from the elevation data using ArcGIS. Before putting in a model run, the resolution of all variables was standardized to 2.5 arc minutes (approximately 5 km) using the “Data Management” tool in ArcGIS.

The future climate variables for the 2050s (2041–2060) and 2090s (2081–2100) were selected from the BCC-CSM2-MR model of the Sixth International Coupling Model Comparison Plan (CMIP6), and the emission path is the shared socioeconomic path (SSP). The BCC-CSM2-MR model has higher resolution in both the atmosphere and the land surface, as well as a more detailed description of the topography. This allows for better simulation of the extreme temperature index and its trend in our study area, which is China, as well as the global land area. Additionally, it can accurately represent the distribution of topographic precipitation and local air temperature (Wu et al., 2019). There are three emission scenarios in SSP1-2.6, SSP-2.45, and SSP5-8.5. Compared with CMIP5, SSPs are not only the latest emission scenarios but also more reliable for the simulation of temperature, precipitation, and its changing trends in China and even global land areas (Wu et al., 2014), and the simulated results also more closely resemble the truly observed results (Fan et al., 2020). Among them, SSP1-2.6 is a low greenhouse gas concentration scenario; SSP2-4.5 is a moderate radiation forcing scenario, representing a compromise carbon emission scenario for medium social vulnerability and moderate forcing (Thomson et al., 2011); in contrast, SSP5-8.5 is a high-forcing climate scenario characterized by high population growth, high carbon emissions, and the most severe trends in global warming (Chen et al., 2022). This study defaults soil and topographic variables to remain unchanged over the projection period, primarily due to the slower change in these factors and the lack of data related to them in future periods (Guo et al., 2019).

To avoid overfitting of the model caused by more environmental factors and affecting the prediction accuracy, we used the relevant code in R 4.1.3 to check the multicollinearity and Pearson’s correlation coefficient among the 38 environmental variables (Dormann et al., 2013), deleted the factor of the correlation coefficient |*r*| ≥0.7 according to the results (Dormann et al., 2013; Feng et al., 2019; Shi et al., 2022; Zhang et al., 2022), and then selected the variance expansion on this basis. Variance inflation factors |VIFs| are <5 factors, and we retained the factors that are generally more important to plant ecology in the excluded factors, to improve the accuracy of the model (Zhao G, et al., 2021). According to the trial operation results of the model, 12 environmental factors were finally selected to participate in modeling, namely, isothermality (Bio3), temperature seasonality (Bio4), max temperature of the warmest month (Bio5), min temperature for the coldest month (Bio6), mean temperature of the wettest quarter (Bio8), annual precipitation (Bio12), precipitation of the wettest month (Bio13), slope, surface soil salt base saturation (T-bs), surface soil exchangeable sodium salt percentage (T-esp), surface soil cation exchange (T-cec soil), and surface soil mucus component cation exchange (T-cec clay).

### 2.2 Modeling procedure

#### 2.2.1 Model construction and evaluation

The effective species distribution sample points and the screened environment variable data were imported into MaxEnt 3.4.4. A quarter of the distribution point data was selected as the testing data, and the remaining data were selected as the training data. The number of replicates was 10, the maximum number of background points was set to 10,000, “bootstrap” was selected as the replicated run type, the grid data were in logistic output format, and the jackknife and create response curves were checked for the simulation.

We used three commonly used assessment metrics—AUC, TSS, and Kappa—to evaluate the accuracy of the model’s prediction results. The area under the receiver operating characteristic curve (ROC) is called the AUC value, which is the best indicator to detect the accuracy of the model so far (Fielding and Bell, 1997). The AUC value ranges from 0 to ~1, and as it tends to be closer to 1, it indicates a better prediction of the model. When the AUC value is less than 0.6, it indicates that the model has bad prediction performance, 0.6~0.7 indicates that the model has poor prediction performance, 0.7~0.8 indicates that the model has fair prediction performance, 0.8~0.9 indicates that the model has good prediction performance, and when the AUC value is more than 0.9, it indicates that the model has excellent prediction performance (Hanley and McNeil, 1982; Araújo et al., 2005). True skill statistic (TSS), which represents the net prediction success including both distributed and undistributed samples, has been widely utilized in various ecological models in recent years (Wang et al., 2022a). The Kappa statistic is used to assess the accuracy of model predictions by determining the percentage of locations where the species’ presence is recorded in the training dataset. It helps determine how well the model’s predicted results agree with the actual results. They both have a range of [−1, +1]. When the value is closer to +1, it means that the model predicts better, while a value less than 0 indicates a more stochastic simulation effect (Allouche et al., 2006).

#### 2.2.2 Model optimization

To avoid overfitting due to the high complexity of the model constructed with the default parameters, which may cause the predicted distribution of the potential habitat of *C. retusus* to deviate too much from the actual situation, this study used the ENMeval data package in R 4.1.3 (Muscarella et al., 2014; Team. R.C., 2014) and adjusted the two most important parameters, namely, regularization multiplier (RM) and feature combination (FC), to improve the prediction accuracy of the model. Among them, FC parameters have five characteristics: linear (L), quadratic (Q), hinge (H), product (P), and threshold (T). We selected six feature combinations used to combine linear—L, LQ, H, LQH, LQHP, and LQHPT—according to the arrangement and combination of 48 parameter combinations (Phillips et al., 2006) and set the RM parameter to 0.5~4, increasing by 0.5 each time, for a total of 8 RM parameters (Elith et al., 2011; Guevara et al., 2018; Zhao G. et al., 2021). Finally, the fitting and complexity of the model are tested according to the minimum information criterion AICc value (delta.AICc) and the difference between the AUC value (avg.diff.AUC) in the test results. The smaller the delta.AICc, the better the model prediction results (Phillips et al., 2017).

### 2.3 Evaluation of environmental variables

According to the prediction results of the MaxEnt model, the percentage contribution (PC), permutation importance (PI), and jackknife test of each environmental factor were combined to evaluate their importance in influencing the distribution of *C. retusus* potential habitat areas, in which the larger the PC value of the environmental factor, the more significant its effect on the distribution of the species. The response curves were plotted against dominant environmental factors to analyze *C. retusus* preference for different environmental factors and climatic characteristics of their potential habitat patches.

### 2.4 Evaluation of current and future potential habitats

#### 2.4.1 Classification of the habitat of *Chionanthus retusus* and its area statistics

The prediction of the potential habitat areas of the species was done based on the MaxEnt model. Firstly, we set the optimized model parameters in the MaxEnt model, and then the mean values of the results were imported into ArcGIS 10.4.1. The tool “Reclassify” was used to classify the suitable zones of *C. retusus* in different periods and climate scenarios (Tang et al., 2022), and the potential suitable zones were divided into four classes using Jenks’ natural breaks method: unsuitable habitat (0~0.1), slightly suitable habitat (0.1~0.24), moderately suitable habitat (0.24~0.5), and optimally suitable habitat (0.5~1). On this foundation, we used ArcGIS for visualization using the raster calculator to count the area of each part of the suitable area under different current and future climate scenarios for comparison and analysis (Zheng et al., 2021).

#### 2.4.2 Changes in the spatial pattern of suitable distribution area for *Chionanthus retusus*

Habitat change is a key factor influencing species distributions. To explore trends in changes in potential habitat loss and increase of *C. retusus* under different climate scenarios, in this study, continuous probability value maps from the MaxEnt model output were classified into binary maps based on “maximum training sensitivity plus specificity” (Yin et al., 2022). Predicted probabilities of presence higher than the threshold value (in this study, the value was 0.24) were assigned 1, representing suitable habitats; the opposite was assigned 0, representing unsuitable habitats (**Figure 1B**). The changes in suitable areas by comparing the suitable/unsuitable distribution maps under the current and future periods were analyzed.

To quantify the loss or increase of potentially suitable areas, we also calculated the percentage change in suitable areas under the two time periods. In addition, the SDMTool packet in R was used to calculate the latitude and longitude of the centers of mass of the suitable area domains under different climate scenarios of *C. retusus*, and the geosphere packet was used to calculate the distance of its center of mass migration. Finally, they were imported into ArcGIS to analyze the direction and magnitude of changes in the suitable areas of *C. retusus* in the future period.

### 2.5 Marxan model construction

The Marxan 4.0.6 model is a systematic conservation planning model based on simulated annealing (SA), which selects the optimal solution from a planning cell of all possible solutions, to achieve optimal conservation at minimum cost, and applies to the problem of planning nature reserves (Moilanen et al., 2009). On the basis of the following steps, we determined the location of the conservation priority area for *C. retusus* under the current period: 1) planning cell and cost setting: we divided China into 10 km × 10 km grids as the planning cells and then used the Zonal Statistics as Table tool in ArcGIS to count the suitable habitat zones for the target species within each planning cell as the cost of conserving each planning cell. 2) Conservation target of the species: The conservation target was set to 30% of the area of total habitat distribution based on the conservation rank of the tree species in China. 3) Marxan run: Boundary length modifier (BLM) is a correction parameter for the length of the boundary of the study area (Tang et al., 2021). Through the modification of the BLM, the cost of the results and the relationship between the total length of the boundary and the total area can be analyzed, to find a point that can balance the two, for iterative calculation to get a more scientific and effective distribution of the protection priority area. In this study, the BLM value is finally selected as 100, and the number of iterations is set to 50. 4) The selected and unselected units of the Marxan model are used as the basis for the division of the systematic protection planning, and the results were imported into ArcGIS to make the priority protection zone planning map of *C. retusus*.

### 2.6 GAP analysis of protected areas for *Chionanthus retusus*

GAP analysis allows for a more general assessment of conservation effectiveness (Rodrigues et al., 2004a). GAP analysis is expressed as the vacancy between the conservation priority areas, covered by nature reserves, and the conservation target set, which is a measure of conservation effectiveness. Areas of conservation priority that are not covered by nature reserves are called conservation vacancy areas. For species, if the percentage of a species’ range covered by nature reserves meets the conservation target, the conservation effect is considered good; conversely, it indicates that there is a conservation vacancy for the species (Rodrigues et al., 2004b). A total of 1,028 nature reserves and 8 national parks were involved in this study, and the data were mainly obtained from the China Nature Reserve Specimen Resource Sharing Platform (http://bhq.papc.cn/specimen.html). Among them are the strictest types of nature reserves in China, which are the main means of biodiversity protection. In order to show the conservation status of *C. retusus* in China in the current period, the systematic conservation planning map of *C. retusus* was overlaid with the vectorial boundaries of China’s wilderness areas in ArcGIS, resulting in a map of conserved vacancy for GAP analysis, which provides a theoretical reference basis for the development of scientific conservation and management of *C. retusus*.

## 3 Results

### 3.1 Model optimization and accuracy evaluation

In this study, based on 131 distribution records and 12 environmental variables, the MaxEnt model after optimizing with the ENMeval package was used to predict the potential adaptive region of *C. retusus*. The optimal combination of parameters was obtained: when delta.AICc = 0, RM = 1.5, and FC = LQH, the delta.AICc value and avg.diff.AUC values at this time were less than the model under the default parameter (**Table 1**), making it clear that this parameter combination can availably decrease the complexity and overfitting of the simulation results and increase the accuracy of the prediction.

**Table 1:** Evaluation results of the MaxEnt model generated by ENMeval.

Model evaluation	Feature combination	Regularization multiplier	delta.AICc	Avg.diff.AUC
Default	LQHPT	1	81.94	0.07802
Optimized	LQH	1.5	0	0.06141

Therefore, we chose the parameter settings of RM = 1.5 and FC = LQH for modeling. After 10 repeated training sets, the average AUC value was 0.960 (**Supplementary Figure S1**). The standard deviation was 0.005, and the average AUC value of the testing set was 0.941, with a standard deviation of 0.016. Both AUC values were above 0.9. The TSS was 0.804, and the maximum Kappa value was 0.62. Both the TSS and Kappa values were greater than 0 and relatively close to +1. So, all three assessment methods showed that the accuracy of using this model to predict the potential suitable habitat distribution of *C. retusus* was excellent.

### 3.2 Evaluation of dominant environmental variables

#### 3.2.1 Impacting the dominant environmental variables on the habitats of *Chionanthus retusus*

**Figure 2:**
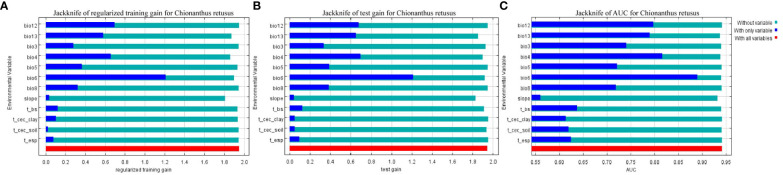
Results of jackknife evaluations of the predictor variables, using regularized training gain **(A)**, testing gain **(B)**, and AUC on the test data **(C)** in the MaxEnt model for *Chionanthus retusus*.

The jackknife method in the MaxEnt model was used to obtain a test diagram of the importance of the distribution of 12 environmental factors to *C. retusus* in the current period (**Figure 2**), and the PC and PI of each environmental variable were calculated by the model, as can be seen from **Table 2**. Bio6, Bio12, and Bio4 were the three variables with the highest PC, with a cumulative contribution rate of 74.2%. Although the contribution rate of Bio13 was only 6.4%, its permutation importance reached to 41.8%, while the PC and PI of other environmental variables were relatively low.

**Table 2:** Percentage contribution and permutation importance of the 12 environmental variables in the MaxEnt model.

Variables	Percentage contribution (%)	Permutation importance (%)	Variables	Percentage contribution (%)	Permutation importance (%)
Bio6	29.8	24.4	T-cec_clay	3.4	1.4
Bio12	24.6	0.3	Bio5	1	1.8
Bio4	19.8	20.6	Bio8	0.4	0.9
Slope	7.6	6.4	Bio3	0.3	0.6
Bio13	6.4	41.8	T-esp	0.3	0.3
T-bs	6.3	1.2	T-cec_soil	0.2	0.3

Further tests based on the jackknife method showed that when only one variable was used for the simulation, the first three variables of regularization training gain (**Figure 2A**), test gain (**Figure 2B**), and AUC (**Figure 2C**) were Bio6, Bio12, and Bio4, showing that these variables contain more effective information than others. When a variable was ignored and the residual variable was used for the simulation, the three variables with the largest reduction in regularized training gain, test gain, and AUC value were T-cec soil, T-esp, and T-cec clay, indicating that these variables had less impact on the prediction of the adaptive area distribution of *C. retusus*.

The comprehensive evaluation showed that the leading environmental factors impacting the distribution of existing potential suitable habitats of *C. retusus* are Bio4, Bio6, Bio12, and Bio13.

#### 3.2.2 Environmental characteristics of the suitable habitat for *Chionanthus retusus*

**Figure 3:**
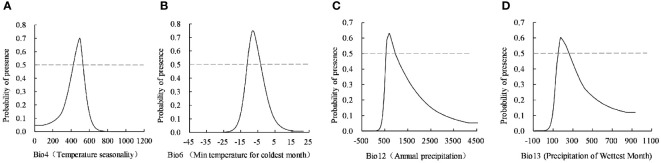
The response curve of four dominant environment variables. **(A)** Temperature seasonality, **(B)** Min tempcrature for coldest month, **(C)** Annual precipitation, **(D)** Precipitation of wettest month.

The MaxEnt model provided the response curve between the probability of species presence and the dominant environmental variables. The habitat with a probability of existence >0.5 was the optimally suitable habitat of the simulated *C. retusus*, and the corresponding environmental variable range was the most suitable for species survival. According to the response curve of four important environmental variables (**Figure 3**), the index value of each dominant environmental variable was dynamically related to its existence probability. Based on the largest percentage contribution of the lowest temperature to the suitable habitat of *C. retusus* in the coldest month, when the temperature was lower than −20°C, it is not suitable for its survival. With the continuous temperature rise, the probability of existence begins to increase significantly until the peak of −8~6°C, being the most suitable growth condition, and the range was −11.21°C to 3.31°C. The other three dominant environmental variables are similar. The probability of the existence of *C. retusus* initially increases with the increase of temperature seasonality, annual precipitation, and precipitation of the wettest month and then decreases with its increase after reaching the peak. The threshold of the most dominant environmental variables affecting the suitable habitats of *C. retusus* according to the response curve was determined as follows: temperature seasonality 902.14~1,088.18, min temperature of the coldest month −11.21~3.31°C, annual precipitation 557.75~955.96 mm, and precipitation of the wettest month 151.06~260.83 mm.

### 3.3 Potential suitable habitats of *Chionanthus retusus* under the current climate condition

**Figure 1A** showed the distribution of potential suitable habitats for *C. retusus* in China under current climatic conditions for the 131 existence points, all basically located in it. Among them, the average suitability of 131 records was 0.55, the maximum suitability was 0.882 (Longquan Town, Yantai City, Shandong Province), and the minimum suitability was 0.010 (Xingning City, Meizhou City, Guangdong Province).

**Supplementary Table S2** showed the area and percentage of the total study zone of potential suitable habitats at each level under current climatic conditions. In this study, total suitable habitat refers to the sum of the moderately and optimally suitable habitat. It was 173.92 × 10^4^ km^2^, accounting for 18.12% of the whole research area. **Figure 1A** showed that the optimally suitable habitat was mainly located in central and eastern China, including southwestern Shanxi, western Hebei, most of Shandong, and northwestern Henan, as well as a small part of southeastern Shaanxi and southern Liaoning. The moderately suitable habitat was mainly concentrated in Shaanxi, Shanxi, Hebei, Shandong, Henan, Anhui, and Hubei. The slightly suitable habitat was mainly located in eastern Sichuan, Chongqing, Guizhou, Hunan, Jiangxi, and Zhejiang. In addition to the above areas, the other areas under the scope of the study were not suitable for the growth of *C. retusus*. They were mainly in most of northeast China, Xinjiang, Gansu, Qinghai, Tibet, and the southernmost areas.

### 3.4 Potential suitable habitat of *Chionanthus retusus* under future climate conditions

**Figure 4:**
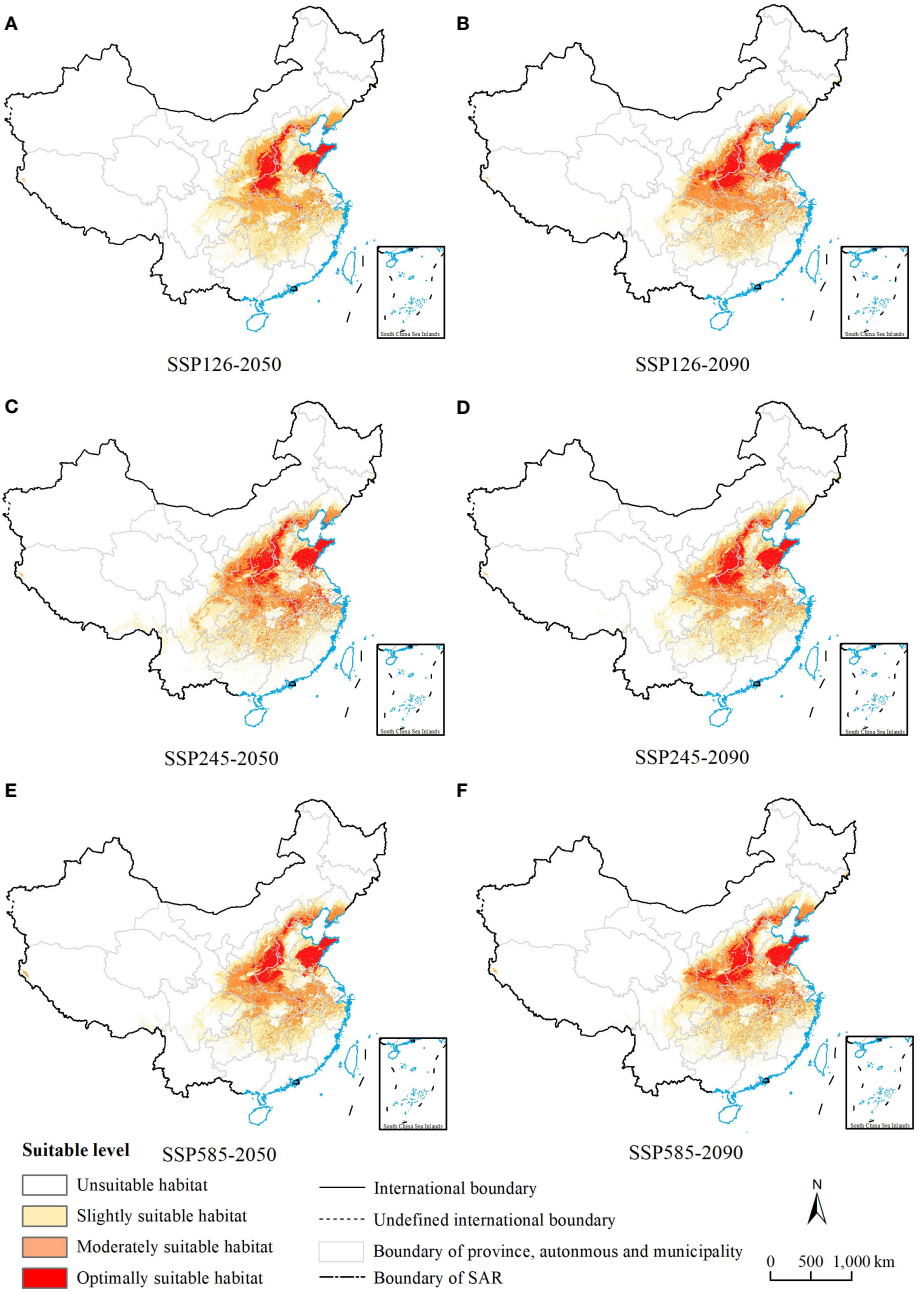
MaxEnt model predictions of the potential suitable habitats of Chionanthus retusus in China for future periods. **(A, C, E)** Three different emission scenarios for ssp1-2.6, ssp2-4.5 and ssp5-8.5 in the 2050s. **(B, D, F)** Three different emission scenarios for ssp1-2.6, ssp2-4.5 and ssp5-8.5 in the 2090s.

**Figure 5:**
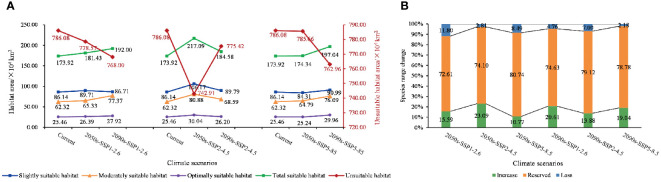
Map of changes in suitable habitat areas for each class. **(A)** Changes in the class of suitable areas at various levels under SSP1-2.6, SSP2-4.5, and SSP5-8.5 climate scenarios. **(B)** Proportions of increase, unchange, and decrease in the area of suitable habitat under different climate scenarios.

In this study, used the MaxEnt model to project the distribution of potential suitable areas for *C. retusus* under the SSP1-2.6, SSP2-4.5, and SSP5-8.5 climate change scenarios for the 2050s and 2090s (**Figure 4**). By comparing the projected distribution of suitable habitats between the next two periods, it can be concluded that there are differences in the increase and loss of suitable areas for each class of *C. retusus* due to climate change impacts (**Supplementary Table S2**; **Figure 5A**).

The area of all levels of suitable habitats increased under future climate conditions compared with the current period, with the largest change in the 2050s under SSP2-4.5, and the increase in area was 43.17 × 10^4^ km^2^, approximately 4.49%. However, with the change in chronology, the most pronounced increase was observed under the SSP5-8.5 condition in the 2090s, and the area was 23.12 × 10^4^ km^2^, approximately 2.40%.

The increase and decrease of the total suitable area of *C. retusus* under the three different climate scenarios varied in time scales (**Figure 5A**). In the SSP1-2.6 emission condition, with the exception of a slight decline in mildly suitable areas from the 2050s to the 2090s, all levels of suitable areas have been on an upward trend, with an increase of 18.08 × 10^4^ km^2^ in total habitat suitable area. In the SSP2-4.5 emission condition, the area of suitable habitats for all classes declined over time after the 2050s but still showed an overall increase compared with the current period. Compared with the first two climatic conditions, the area of all suitable zones under SSP5-8.5 showed a steady increase over time. Thus, under the three climatic conditions, the increase in total suitable habitat area was most significant under SSP5-8.5, which was 32.51 × 10^4^ km^2^.

### 3.5 Dynamic changes of suitable habitats under different climatic scenarios at different periods

**Figure 6:**
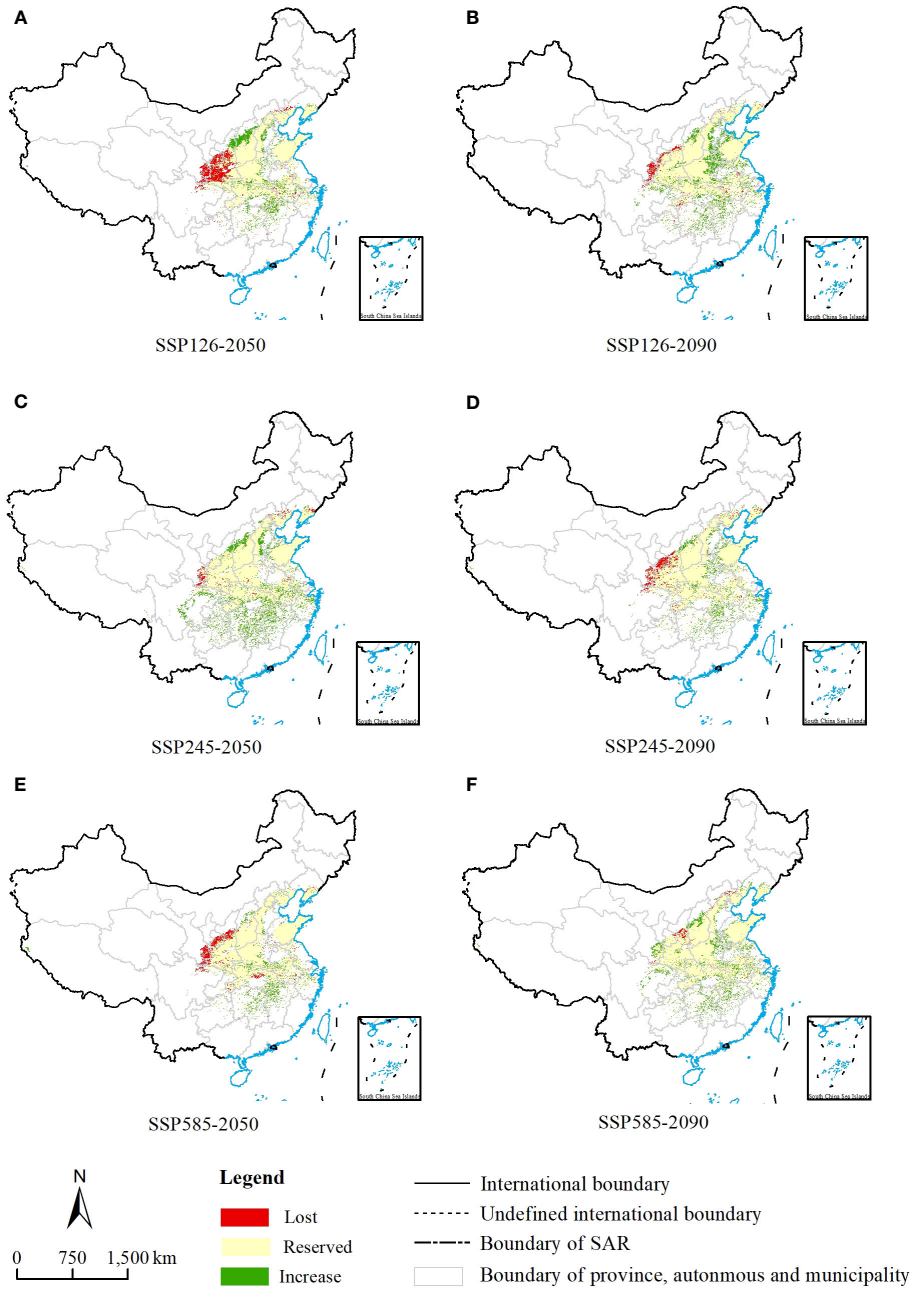
Change in the suitable/unsuitable habitats of Chionanthus retusus under the 21st-century climate change. **(A, C, E)** Three different emission scenarios for ssp1-2.6, ssp2-4.5 and ssp5-8.5 in the 2050s. **(B, D, F)** Three different emission scenarios for ssp1-2.6, ssp2-4.5 and ssp5-8.5 in the 2090s.

Based on a map of current suitable habitat distribution and a map of suitable habitats projected by simulation under different climate scenarios for future periods, we mapped the range shifts in potential suitable habitats of *C. retusus* in the future (**Figure 6**). Based on **Supplementary Table S3** and **Figure 5B**, it can be concluded that *C. retusus* had the largest percentage of unchanged potential habitat area, and all of the increased area was greater than the percentage of lost area, but the degree of change varied across climate scenarios. The most significant increase in regional share was in the 2050s under the SSP2-4.5 scenario (23.09%), while the most significant loss was in the 2050s under SSP1-2.6 (11.80%). Based on **Figure 6**, it can be seen that the areas of increase in suitable areas were mainly located in the central part of Shanxi and the northern part of Henan, as well as in Hubei, Chongqing, Hunan, Jiangxi, and Zhejiang having sporadic distribution. The areas of loss were concentrated in the southern part of the three provinces of Shaanxi, Ningxia, and Gansu. The results suggest that climate change in the 21st century threatens the western part of the current *C. retusus* suitability habitat, while the southern part will benefit from climate change.

**Figure 7:**
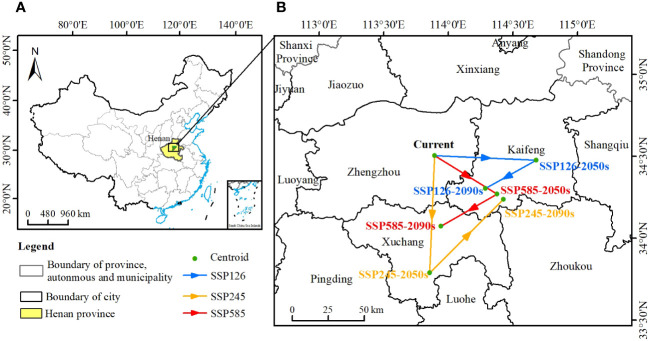
The centroids of the suitable habitats of *Chionanthus retusus*. **(A)** The location of the regions with centroid changes in China. **(B)** Localized enlargement of **(A)**.

The geographic centroids of the potential habitat of *C. retusus* were different for different periods and climate change scenarios, and it was mainly located in the Henan Province of China (**Figure 7A**). Looking at the details (**Figure 7B**), the geographic centroid of the modern potential habitat was located in Xinzheng City, Zhengzhou City, Henan Province (34.62°N, 113.81°E). When the climate scenario was SSP126-2090s, the centroid shifted to the southwest and was located in Yushi County, Kaifeng City, Henan Province (34.39°N, 114.18°E), and the range shift was 42,220 m. When the climate scenario was SSP245, the centroid shifted to the south in the 2050s and was located in Xiangcheng County, Henan Province (33.91°N, 113.70°E), and then transferred back to Yushi County, Kaifeng City, Henan Province (34.31°N, 114.30°E) in the 2090s, and the range shift was 72,318 m. When the climate scenario was SSP585-2090s, the centroid of *C. retusus* was transferred southward, and it was located in Changge City, Xuchang City, Henan Province (34.19°N, 113.81°E), and the range shift was 45,257 m. In summary, the centroid of *C. retusus* was located in Changge City, Xuchang City, Henan Province, with a shift range of 45,257 m. In a word, the centroid of the potential suitable habitats of *C. retusus* in China will range southward under the 21st-century climate change.

### 3.6 Priority protected areas of *Chionanthus retusus* under current climate conditions

**Figure 8:**
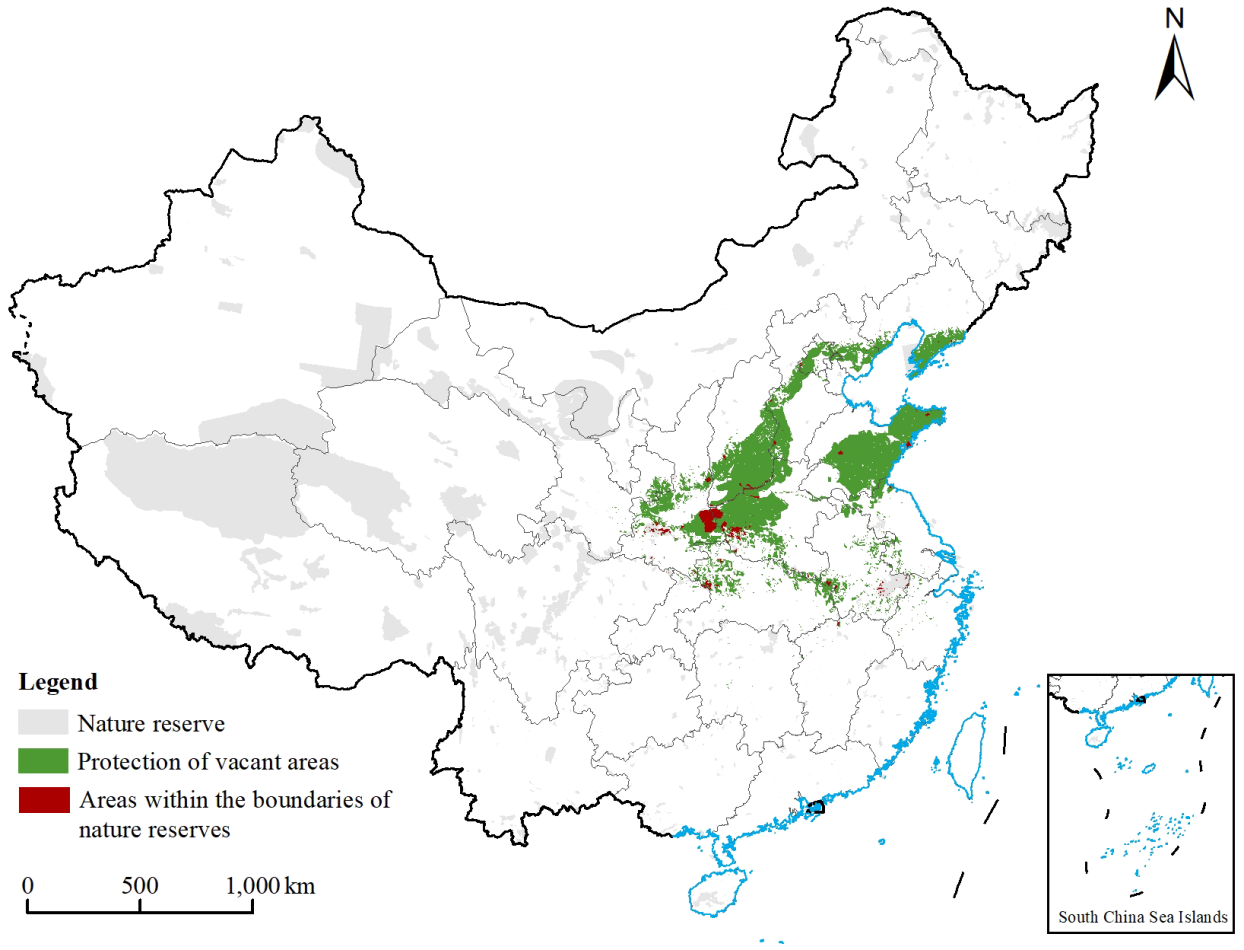
Distribution of each type of protected area range in China. The gray area indicates the range of nature reserves and national parks in China in the current period, the green area indicates the range of *Chionanthus retusus* priority protected areas, and the red area indicates the range of vacant areas in *C. retusus* priority protected areas.

We used the Marxan model to calculate the priority conservation zones of *C. retusus*, and the results were imported into ArcGIS to construct a systematic conservation planning map with *C. retusus* as the main conservation target (**Figure 8**). The map indicated that the priority conservation areas of the species were concentrated in Shandong, southwestern Shanxi, southwestern Hebei, and north-central Hubei, which was consistent with the medium-height suitability area of *C. retusus* predicted by the Maxent model, explaining that the prediction results were resultful. Moreover, the area of the systematic conservation plan and the location of regional distribution show that the priority conservation area of *C. retusus* is 38.50 × 10^4^ km^2^, which accounts for approximately 4.01% of the total land area of China, and the relatively concentrated distribution and the small proportion of land area make it easier to develop specific protection and management measures.

### 3.7 Protective effects of priority conservation areas for *Chionanthus retusus*

The protection of *C. retusus* had obvious gaps in the premise of existing protected areas (**Figure 8**). The results showed that the area of nature reserves in China is 126.54 × 10^4^ km^2^, of which the area of the part overlapping with the *C. retusus* priority protected areas was 1.93 × 10^4^ km^2^, and the area of the vacant priority protected areas was 36.57×10^4^ km^2^, with approximately 94.98% of the preferred areas of protection not currently designated as protected areas. The areas of vacancies were mainly concentrated in most of Shandong, southern Liaoning, southwestern Shanxi, western Henan, and central Hebei, as well as a small distribution in the provinces of Shaanxi, Hubei, and Anhui. Based on the existing protected area protection units, by comparing the existing wetland protected areas in the country as well as the results of the protected area preference, it was determined that the protected vacant areas of *C. retusus* are much larger than the protected areas, indicating that there are many areas suitable for its growth that have not been protected.

## 4 Discussion

### 4.1 Effects of dominant environmental variables on the potential suitable habitat of *Chionanthus retusus*

This study evaluated the importance of each environmental factor by integrating the PC and PI of each environmental variable factor and jackknife, indicating that the temperature seasonality, min temperature for the coldest month, annual precipitation, and precipitation of the wettest month were the most dominant environmental variables affecting the potential suitable habitats of *C. retusus*. The integration results showed that the medium-optimally suitable habitat of *C. retusus* is mainly distributed in semi-humid monsoon climate areas with an annual precipitation of 600~1,000 mm and approximately 25~35°N in China. Its growth characteristics are consistent with the leading environmental factors that influence the growth of *C. retusus*.

The suitable habitats of plants on Earth are greatly limited by climatic conditions (Zhang et al., 2022). By comparing the distribution and changes in suitable habitats of *C. retusus* by class in current and future periods and observing the trajectory of its shifts of the geographic centroids, *C. retusus* may be better suited to climatic conditions under the higher concentration emission scenarios, as evidenced by the fact that the total suitable habitat area under the different climate scenarios increased compared with the current period. Moreover, the increased area of the optimally suitable habitat is concentrated in southern regions such as Hubei and Anhui, including the overall shift of the centroid. Liu et al. (2010) have shown that the seasonal change intensity of the climate affects the distribution of vegetation. As a slow and long-lived woody plant, the seasonal change of *C. retusus* in a year is the key factor affecting its potential geographical distribution. Although *C. retusus* is widely distributed in China, its development is slow, it has a long maturation cycle, and the hermaphrodite is difficult to pollinate. Moreover, its seed has the dormancy of the epicotyl of a typical ornamental plant (Fan et al., 2016), resulting in a long time for rooting and germination, and the seed has high requirements for ambient temperature and humidity. This characteristic means that after the seeds have been rooted, their epiblast must be subjected to a low temperature of 1~10°C before the seedlings can grow out of the ground. It was also found through our experimental results that *C. retusus* was very sensitive to the response to the lowest temperature in the coldest month, which showed that the low-temperature limit demand is likely to be an important factor in the overall southward range shift of the suitable habitat, but significant warming has led to an increase in the lower limit of minimum temperatures, resulting in their seeds not being able to germinate properly without access to cooler temperatures. This is also one of the main reasons for the dramatic decline in the total suitable habitat area in the 2050s under SSP2-4.5. Mu and others (Mu et al., 2022) have shown that *C. retusus* has a certain ability to regulate the flooded environment, but they can only reach the degree of wet damage and cannot withstand the waterlogging environment. The response curve obtained in this study showed that when the presence probability of *C. retusus* is >0.5, the suitable annual precipitation is 557.75~955.96 mm, and the wettest monthly precipitation is 151.06~260.83 mm. When the precipitation exceeds the threshold, the probability of existence will drop rapidly, which is not conducive to the survival of the tree species. Therefore, *C. retusus* mostly grows in mountainous and hilly areas with a certain slope, which can reduce the water accumulation time and prevent its ventilation. The tissue turns black and decays, leading to death.

### 4.2 Conservation status of *Chionanthus retusus* in the current period and recommendations for future planning of protected areas

Overall, it seemed that only 4.01% of *C. retusus* conservation priority zones are protected by nature reserves in China. By comparing the results of the *C. retusus* conservation unit preferences with the existing conservation pattern in China, the results of the conservation gap analysis indicate that there are still large gaps in the conservation of the study area. *Chionanthus retusus* is a native tree species with high comprehensive utilization value and development potential in China, especially in the field of medicine, which is extremely useful, and its flowers contain flavonoids with good antimicrobial effects, which can be used to treat a variety of inflammatory and neurological disorders (Lee et al., 2019; Wang et al., 2022b). However, as a result of *C. retusus*’s own genetic mode and long-term selection with the environment in the phylogenetic process, it has caused its ecological amplitude to become narrower, and its ability to reproduce on its own has been reduced, which is one of the important reasons why rare and endangered species are easy to be reduced in the current situation. Thus, optimizing and adjusting nature reserves based on the results of the conservation priority areas in this study can serve as a scientific reference for the construction of grids of protected areas for *C. retusus* in the future, which will provide a foundation for conducting conservation of endangered species following feasibility studies.

### 4.3 Limitations of the models’ predictions in future periods and further outlooks

In this study, on the one hand, only the bioclimatic factors of the corresponding period were used in predicting the potential suitable habitats under different climate scenarios in the future, and since soil and topographic data for the future are difficult to obtain, the default is unchanged from the present. However, in reality, soil property factors such as soil salinity saturation and topographic factors such as slope also affect their potential geographic distribution. On the other hand, this study only considered the two conservation conditions of nature reserves and national parks when exploring systematic conservation planning, and other conservation conditions have not yet been considered. Therefore, we know that the simulation results for the suitable habitat in the future period have some limitations, and the local vegetation cover data, geological conditions, human activities, and other influencing factors need to be considered in the subsequent practical applications. For species like *C. retusus*, which has high economic value, human activities and regional economic conditions should be considered as predictive factors for its impact. Moreover, when establishing a protected area, it is important to take into account whether the area is a densely populated urban area or other specific circumstances that make it unsuitable for the establishment of a nature reserve. In addition, this study only explored the distribution of *C. retusus* and its changes in the suitable habitat in China, which has certain regional limitations. In the future, we can also gather data on the distribution of *C. retusus* in other regions and even worldwide, in order to conduct a more comprehensive study of the suitable environmental conditions that are conducive to the growth of *C. retusus*. However, the results of this study provide some theoretical support for the development of reasonable adaptation strategies for rare native tree species in response to climate change and are still of great significance in guiding the conservation of germplasm resources, future planting planning, and sustainable development and utilization of *C. retusus*.

## 5 Conclusion

We used the optimal model with RM = 1.5 and FC as LQH to predict the potential suitable habitat of *C. retusus* under three climate scenarios (SSP1-2.6, SSP2-4.5, SSP5-8.5) in the current and future periods (2050s and 2090s), to analyze the changes in its spatial pattern under the 21st-century climate change, and we also used the Marxan model and gap analysis to simulate its priority conservation areas and assess the current state of conservation. These results showed that precipitation (Bio12, Bio6, and Bio13) and temperature (Bio4) play important roles in the distribution of habitats of *C. retusus*. As climate change intensified, the total suitable area of *C. retusus* increased to varying degrees in the 21st century, with an overall expansion of the optimally suitable habitats to the south, and warming had a positive effect on their distribution to a certain extent. However, it was found that there is still a big gap in the current protection of *C. retusus* priority conservation areas. So, these results improved the scientific theoretical reference for the subsequent research on the species distribution pattern and the introduction of cultivation and systematic protection planning of more national key protected species and high economic value plants.

## Data availability statement

The raw data supporting the conclusions of this article will be made available by the authors, without undue reservation.

## Author contributions

YW: Writing – original draft, Formal analysis, Investigation, Methodology, Supervision, Writing – review & editing. KW: Formal analysis, Methodology, Resources, Visualization, Writing – original draft, Writing – review & editing. RZ: Data curation, Investigation, Writing – review & editing. LX: Data curation, Investigation, Writing – review & editing. YL: Data curation, Investigation, Writing – review & editing. GZ: Formal analysis, Methodology, Software, Writing – review & editing. FZ: Funding acquisition, Supervision, Writing – review & editing.
