# Supplementary material for: Prediction of potential suitable habitats in the 21st century and GAP analysis of priority conservation areas of Chionanthus retusus based on the MaxEnt and Marxan models
Source: Front Plant Sci. 2024 Feb 29;15:1304121. doi: 10.3389/fpls.2024.1304121 (PMC10937578; doi:10.3389/fpls.2024.1304121)
Supplement: Supplementary file 1 [file DataSheet_1.pdf]

Supplementary Material

Prediction potential suitable habitats in the 21st century and GAP analysis of priority conservation areas of Chionanthus retusus based on Maxent model and Marxan model

Yongji Wang[1+], Kefan Wu[1+], Ruxia Zhao[1], Liyuan Xie[1], Yifan Li[1], GuanghuaZhao[2*], Fenguo Zhang[1*]

[1]School of Life Science, Shanxi Engineering Research Center of Microbial Application Technologies, Shanxi Normal University, Taiyuan, Shanxi, China

[2]Administrative Office, Shanwei Middle School, Shanwei, China

Supplementary Table 1. Environmental variables used in MaxEnt model, the bolded variables were those that remain after screening to participate in modeling.

Category	Variable	Description	Unit
Climate	Bio1	Annual Mean Temperature	℃
	Bio2	Mean Diurnal Range	℃
	**Bio3**	**Isothermality**	**/**
	**Bio4**	**Temperature Seasonality**	**/**
	**Bio5**	**Max Temperature of Warmest Month**	**℃**
	**Bio6**	**Min Temperature of Coldest Month**	**℃**
	Bio7	Temperature Annual Range	℃
	**Bio8**	**Mean Temperature of Wettest Quarter**	**℃**
	Bio9	Mean Temperature of Driest Quarter	℃
	Bio10	Mean Temperature of Warmest Quarter	℃
	Bio11	Mean Temperature of Coldest Quarter	℃
	**Bio12**	**Annual Precipitation**	**Mm**
	**Bio13**	**Precipitation of Wettest Month**	**Mm**
	Bio14	Precipitation of Driest Month	Mm
	Bio15	Precipitation Seasonality	/

	Bio16	Precipitation of Wettest Quarter	Mm
	Bio17	Precipitation of Driest Quarter	Mm
	Bio18	Precipitation of Warmest Quarter	Mm
	Bio19	Precipitation of Coldest Quarter	Mm
Soil	**T-bs**	**Base saturation**	**%**
	**T-esp**	**Percentage of exchangeable sodium salt**	**%**
	T-clay	Percentage of clay in topsoil	%
	**T-cec soil**	**Cation exchange capacity**	**Mmol/kg**
	**T-cec clay**	**Cation exchange capacity of clay particles**	**Mmol/kg**
	T-$CaSO_4$	Sulfate content	g/m^3
	T-ece	Electrical conductivity in topsoil	mS/m
	T-$CaCO_3$	Carbonate or lime content	%
	T-gravel	Volume percentage of gravel	%
	T-oc	Percentage of organic carbon	g/kg
	T-pH	pH Value of top soil (H_2O)	/
	T-ref bulk	Soil bulk density	g/cm^3
	T-sand	Surface sand content	g/cm^3
	T-silt	Percentage silt in topsoil	%
	T-teb	Total exchangeable salt bases	Mmol/kg
	texture	Soil property	/
	T-ref bulk	Soil bulk density	g/cm^3
	T-sand	Surface sand content	g/cm^3
Topography	Ele	Elevation	m
	Aspect	Aspect	°
	**Slope**	**Slope**	°

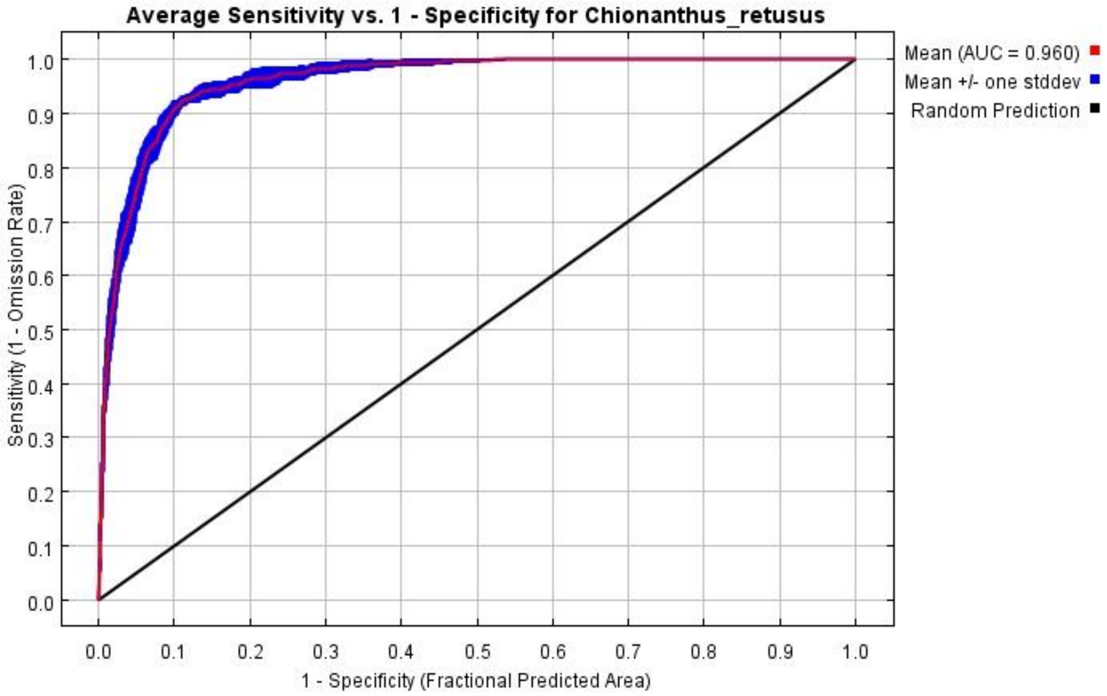

Supplementary Figure 1. ROC curve of the optimized model

Supplementary Table 2. Area statistics of suitable habitats of *C. retusus* under different climate scenarios

Period		Unsuitable habitat	Slightly suitable habitat		Moderately suitable habitat		Optimally suitable habitat		Total suitable habitat	
			10^4/km^2	/%	10^4/km^2	/%	10^4/km^2	/%	10^4/km^2	/%
Current		786.08	86.14	8.97%	62.32	6.49%	25.46	2.65%	173.92	18.12%
2041-2060 (2050s)	SSP1-2.6	778.57	89.71	9.34%	65.33	6.81%	26.39	2.75%	181.43	18.90%
	SSP2-4.5	742.91	106.17	11.06%	80.88	8.42%	30.04	3.13%	217.09	22.61%
	SSP5-8.5	785.66	84.31	8.78%	64.79	6.75%	25.24	2.63%	174.34	18.16%
2080-2100 (2090s)	SSP1-2.6	768.00	86.71	9.03%	77.37	8.06%	27.92	2.91%	192.00	20.00%
	SSP2-4.5	775.42	89.79	9.35%	68.59	7.14%	26.20	2.73%	184.58	19.23%
	SSP5-8.5	762.96	90.99	9.48%	76.09	7.93%	29.96	3.12%	197.04	20.52%

Supplementary Table 3. Changing situation of suitable area of *C. retusus* under current and future climatic condition

Period	Climate scenario	Suitable habitat	Species range change	Loss		Reserved		Increase	
		10^4/km^2	/%	10^4/km^2	/%	10^4/km^2	/%	10^4/km^2	/%
Current		87.78							
2041-2060 (2050s)	SSP1-2.6	91.72	27.40	10.83	11.80	66.59	72.61	14.30	15.59
	SSP2-4.5	110.92	25.90	3.12	2.81	82.19	74.10	25.61	23.09
	SSP5-8.5	90.02	19.26	7.64	8.49	72.68	80.74	9.70	10.77
2080-2100 (2090s)	SSP1-2.6	105.30	25.37	5.02	4.76	78.58	74.63	21.70	20.61
	SSP2-4.5	94.80	20.88	6.63	7.00	75.00	79.12	13.16	13.88
	SSP5-8.5	106.05	21.22	2.32	2.18	83.54	78.78	20.19	19.04
